# Thermoresponsive PNIPAM Gel-Coated PCL Nanofibers: Characterization, Vapor-Phase Release of Rosemary Essential Oil, and Activity Against *Aspergillus flavus*

**DOI:** 10.3390/foods15203757

**Published:** 2026-10-21

**Authors:** Elif Yener, Canan Yagmur Karakas, Fatih Bozkurt, Mehmet Murat Ozmen, Hayrettin Ozer, Ayse Karadag

**Affiliations:** 1 Department of Food Engineering, Faculty of Chemical and Metallurgical Engineering, Yildiz Technical University, Istanbul 34210, Türkiye; 2 Department of Bioengineering, Faculty of Chemical and Metallurgical Engineering, Yildiz Technical University, Istanbul 34210, Türkiye; ozmenm@yildiz.edu.tr; 3 TUBITAK Marmara Research Center (MAM), Kocaeli 41470, Türkiye

**Keywords:** electrospinning, poly(*N*-isopropylacrylamide), antifungal activity, lower critical solution temperature (LCST), headspace analysis

## Abstract

Rosemary essential oil (REO) was incorporated into electrospun poly(ε-caprolactone) (PCL) nanofibers, which were subsequently modified with a thermoresponsive poly(*N*-isopropylacrylamide) (PNIPAM)-gel coating using an immersion-based approach. The coated fibers exhibited temperature-dependent surface wetting behavior below and above the lower critical solution temperature (LCST) of PNIPAM. PNIPAM coating reduced water vapor transmission at 25 °C and increased the water vapor transmission ratio (WVTR_40_/WVTR_25_) from 1.65 to 2.31, whereas thickness-normalized water vapor permeability did not show a distinct LCST-dependent transition. Headspace analysis by HS-SPME-GC-MS showed increasing accumulation of REO volatiles with incubation time and temperature. After 48 h, PNIPAM coating reduced the total headspace amount of the selected volatile markers by approximately 7.2-fold at 25 °C and 4.9-fold at 40 °C. A significant coating × temperature interaction (*p* < 0.05) indicated that the coating modified the temperature dependence of volatile transfer, with the 40/25 °C ratio increasing from 1.65 in uncoated fibers to 2.41 in coated fibers. In non-contact antifungal assays against toxigenic *Aspergillus flavus*, REO-loaded fibers produced transient growth inhibition at 25 °C, whereas inhibition persisted throughout the 10-day incubation at 40 °C and was greater for the uncoated REO-loaded fibers. None of the fiber systems significantly affected AFB_1_ accumulation at 25 °C, while AFB_1_ was undetected in all samples, including the untreated control, at 40 °C. Overall, the PNIPAM-gel coating provided substantial retention of REO volatiles while introducing temperature-dependent modulation of their transfer to the gas phase, although the response did not represent a simple LCST-triggered release switch.

## 1. Introduction

Filamentous fungi are major causes of spoilage in agricultural commodities and stored foods, particularly under warm and humid conditions, resulting in quality losses and reduced shelf life [1]. Of particular concern are toxigenic species such as *Aspergillus flavus*, which can colonize cereals, nuts, and spices and produce aflatoxins [2]. Among these, aflatoxin B_1_ (AFB_1_) is the most significant and is classified as a Group 1 carcinogen by the International Agency for Research on Cancer (IARC) [3]. Concerns over fungal spoilage and mycotoxin contamination, together with increasing demand for alternatives to synthetic preservatives, have therefore stimulated interest in natural antifungal strategies capable of controlling toxigenic fungi in foods [1].

Plant essential oils (EOs) have received considerable attention as natural antifungal agents because of their antimicrobial activity and volatile character, which enables inhibition without direct contact with the food surface [4,5]. Rosemary (*Rosmarinus officinalis* L.) essential oil (REO), rich in monoterpenes such as 1,8-cineole, camphor, and α-pinene, is particularly promising, with inhibitory effects against *A. flavus* growth and aflatoxin biosynthesis previously reported [5]. However, the high volatility and sensitivity of EOs to environmental factors can result in rapid losses of active compounds and limit the persistence of their antifungal activity [4]. Strategies capable of retaining these volatile compounds while controlling their transfer to the surrounding atmosphere are therefore of interest for active food-packaging applications.

Electrospinning provides a versatile approach for incorporating EOs into polymeric carriers, offering high surface area, porous structures, and processing conditions suitable for volatile compounds [6,7,8]. Poly(ε-caprolactone) (PCL), a biodegradable and readily electrospinnable polymer, has been widely investigated as a carrier for EOs in active food-packaging systems [9,10,11]. Beyond conventional encapsulation, surface modification of electrospun fibers provides an opportunity to further regulate the transport of incorporated active compounds [11]. In this context, poly(*N*-isopropylacrylamide) (PNIPAM) is of particular interest because it undergoes a reversible transition at a lower critical solution temperature (LCST) of approximately 32 °C. Below the LCST, PNIPAM is hydrated and hydrophilic, whereas above it the polymer undergoes dehydration and chain collapse, producing marked changes in its interfacial and transport properties [12]. These properties have led to the application of PNIPAM-based systems for temperature-responsive delivery, including emerging applications in active food packaging [13,14,15].

The LCST of PNIPAM also falls within a temperature region relevant to *A. flavus*, although fungal growth and aflatoxin biosynthesis show different temperature dependencies. Mycelial growth is favored at approximately 32–33 °C, whereas aflatoxin production generally occurs over a lower and narrower temperature range and declines at higher temperatures [16,17]. This proximity makes PNIPAM an interesting candidate for investigating whether temperature-responsive surface modification can influence the transfer of antifungal volatiles.

PNIPAM can be incorporated into electrospun systems by blending with the polymer feed solution [17], as a coaxial shell [13], or through post-spinning surface modification [12,18]. Previous thermoresponsive polymer-based systems are summarized in Table 1 to compare the fabrication approaches reported in the literature and to highlight the distinction of the present system. Although PNIPAM-based systems containing EOs have previously been investigated, their release behavior has commonly been evaluated in liquid media, while the temperature-dependent transfer of EO constituents into the headspace from these systems has rarely been quantified. Moreover, information on the antifungal and antiaflatoxigenic performance of such thermoresponsive systems under non-contact conditions remains limited.

In this study, a thermoresponsive PNIPAM coating was applied to preformed REO-loaded electrospun PCL fibers using a relatively simple immersion-based approach. The influence of PNIPAM modification on the temperature-dependent transfer of REO volatiles into the surrounding atmosphere was investigated at 25 and 40 °C, below and above the LCST of PNIPAM, respectively. The resulting fibers were also characterized in terms of their physicochemical properties, temperature-dependent surface wettability, and water vapor transport behavior. Finally, antifungal activity against *A. flavus* and the effect on AFB_1_ accumulation were evaluated under non-contact conditions.

## 2. Materials and Methods

### 2.1. Materials

PCL (Sigma-Aldrich, St. Louis, MO, USA; Mn: 80,000), N-isopropylacrylamide (NIPAM, Tokyo Chemical Industry Co., Ltd., Tokyo, Japan), N,N′-methylenebisacrylamide (BAAm, Bio Basic Inc., Markham, ON, Canada), ammonium persulfate (APS, Sigma-Aldrich, St. Louis, MO, USA), N,N,N′,N′-tetramethylethylenediamine (TEMED), chloroform, and N,N-dimethylformamide (DMF) (ISOLAB Chemicals, Eschau, Germany) were used as received. α-Pinene (98%) and camphor (analytical standard, ≥99.0%, GC) were obtained from Sigma-Aldrich (St. Louis, MO, USA), and 1,8-cineole (≥98.0%, GC) from Merck (Darmstadt, Germany); these were used for GC-MS calibration. Dried rosemary (*Rosmarinus officinalis* L.) leaves were purchased from Arifoglu (Istanbul, Turkiye). Potato dextrose agar (PDA; Merck Millipore, Darmstadt, Germany) and yeast extract sucrose (YES) medium were used for fungal cultivation. A toxigenic *Aspergillus flavus* isolate from the culture collection of TUBITAK MAM Food Institute (Gebze, Turkiye) was used throughout the study.

### 2.2. Extraction and Characterization of REO

#### 2.2.1. Hydrodistillation

REO was obtained from dried rosemary leaves by Clevenger-type hydrodistillation in distilled water (1:6, *w*/*v*) for 3 h [28]. The collected oil was dried over anhydrous sodium sulfate, and stored at 4 °C until further use.

#### 2.2.2. GC-MS Characterization of REO Composition

The chemical composition of REO was determined by gas chromatography–mass spectrometry (GC-MS) according to the method described in [29]. REO was diluted in chloroform, and injected (2 µL) in splitless mode into a GC-MS system (GCMS-QP2010 Ultra, Shimadzu, Kyoto, Japan) equipped with an Rxi-5Sil MS capillary column (30 m × 0.25 mm × 0.25 µm). Helium was used as the carrier gas at a constant flow rate of 1 mL min^−1^, and the injector temperature was set at 250 °C. The oven temperature was initially held at 45 °C for 5 min and then increased to 250 °C at 7 °C min^−1^. The mass spectrometer was operated in electron ionization (EI) mode at 70 eV over a *m*/*z* range of 50–500. REO constituents were tentatively identified by comparison of their mass spectra with the Wiley mass spectral library, supported by their retention times (RTs) and characteristic fragment ions (*m*/*z*). Compounds with a similarity index (SI) below 90% were classified as unidentified, and the relative abundance of each tentatively identified compound was expressed as a percentage of the total chromatographic peak area.

### 2.3. Preparation of PNIPAM Hydrogel-Coated Nanofibers

#### 2.3.1. Empty and REO-Loaded PCL Nanofibers

A 15% (*w*/*v*) PCL solution was prepared in chloroform: N,N-dimethylformamide (DMF) (3:2, *v*/*v*) [30], and stirred at room temperature for 2 h until complete dissolution. The solution was electrospun using an electrospinning unit (Holmarc Opto-Mechatronics Pvt. Ltd., Kochi, India; model HO-NFES-0434) at 17 kV, a flow rate of 0.2 mL h^−1^, and a tip to collector distance of 11 cm using a 23-gauge stainless-steel needle (SAI Infusion Technologies, Lake Villa, IL, USA). Electrospinning was performed at room temperature and approximately 50% relative humidity. REO was incorporated into the polymer solution under ice-bath conditions (0–4 °C) to minimize volatilization. REO-loaded PCL nanofibers with different oil loading levels were prepared using different PCL/REO ratios and electrospinning conditions to obtain continuous and uniform fibers [7]. The formulation compositions and process parameters are summarized in Table 2.

#### 2.3.2. Synthesis of PNIPAM Hydrogel

PNIPAM hydrogel was synthesized via free-radical polymerization of NIPAM (12.5% *w*/*v*) using BAAm (1.32% *w*/*v*), TEMED (2.5% *v*/*v*), and APS (0.8% *w*/*v*) as crosslinker, accelerator, and initiator, respectively [31]. The stock solutions were mixed at a volumetric ratio of 4:1:1 (NIPAM/BAAm/TEMED) and diluted with deionized water. After nitrogen purging at room temperature, APS was added to initiate polymerization.

#### 2.3.3. PNIPAM Gel-Coated Nanofibers

PNIPAM gel coating of the nanofibers was performed using an immersion-based method adapted from [26]. After APS addition, the PNIPAM gel precursor solution was stirred for 30 s, and the nanofibers were immersed in the reactive mixture for different periods (30–120 s) before gelation was completed. The samples were then removed and air-dried at room temperature for 24 h before further analysis [32]. Uncoated fibers were also kept under the same conditions. The nomenclature of the prepared samples was summarized in Table 3.

### 2.4. Characterization

#### 2.4.1. Field Emission Scanning Electron Microscopy (FESEM)

The surface morphology of the nanofibers was examined using a FESEM (Apreo 2S, Thermo Fisher Scientific, Hillsboro, OR, USA) at an accelerating voltage of 10 kV after gold sputter-coating. Fiber diameters were determined from the FESEM micrographs using ImageJ software (version 1.53, National Institutes of Health, Bethesda, MD, USA) by measuring 100 randomly selected fibers per sample. Results were expressed as mean ± standard deviation. The elemental composition of REO-free control fibers, PCL-0 (uncoated) and PCL-PN90 (PNIPAM-coated REO-free fiber), was analyzed by energy-dispersive X-ray spectroscopy (EDS) at 10 kV, focusing on carbon, nitrogen and oxygen.

#### 2.4.2. FTIR and Thermal Analysis

FTIR spectra were recorded using an ATR-FTIR spectrometer (Bruker Tensor 27, Bruker, Karlsruhe, Germany) over the range of 4000–600 cm^−1^ at a resolution of 4 cm^−1^ [33]. Thermal analysis was performed using a DSC (DSC Q20, TA Instruments, New Castle, DE, USA). Approximately 7–8 mg of each sample were hermetically sealed in aluminum pans and analyzed at a heating rate of 1 °C min^−1^ under a nitrogen flow of 20 mL min^−1^ [34]. The temperature range was 0–250 °C for the nanofibers and their components and 20–65 °C for PNIPAM gel and PNIPAM-containing samples.

#### 2.4.3. Contact Angle Measurement

Dynamic contact angles were measured by the sessile drop method using a goniometer (Biolin Scientific, Gothenburg, Sweden) with samples pre-equilibrated at 25 °C and 40 °C, below and above the LCST of PNIPAM [35]. Deionized water was also equilibrated to the corresponding temperature before use. A fixed-volume water droplet (4 µL) was deposited onto the sample surface, and contact angles were recorded at 0, 3, 5, and 10 s. Samples were fixed on a metal plate during pre-equilibration at 40 °C and transferred immediately to the goniometer on the same plate to minimize heat loss.

#### 2.4.4. Mechanical Properties

The mechanical properties of the nanofibers were evaluated using a texture analyzer (TA-XT2Plus, Stable Micro Systems, Surrey, UK) equipped with a 5 kg load cell. Samples were cut into strips (60 × 10 mm). Prior to mechanical testing, the samples were conditioned at 25 °C and 50% RH for 24 h. The samples were then tested at ambient temperature with a gauge length of 50 mm and a crosshead speed of 60 mm min^−1^ [36]. The thickness of each nanofiber mat was measured at three different positions using a Digimatic Micrometer (Mitutoyo, Kawasaki, Japan). The mean thicknesses of PCL-0, PCL-L1, PCL-PN90, and REO-PN90 were 35.44 ± 4.13, 42.89 ± 2.78, 46.33 ± 4.35, and 53.33 ± 3.78 µm, respectively. Tensile strength (TS) and elongation at break (EAB %) values were calculated according to Equations (1) and (2), respectively:

(1) $$\mathrm{TS}\left(\mathrm{MPa}\right)=\left(\frac{\mathrm{RE}\times g}{T\times W}\right)\times 10^{-6}$$

(2) $$\mathrm{EAB}(\%)=\left(\frac{L-L_{0}}{L_{0}}\right)\times 100$$

where T is the sample thickness (m), W is the sample width (m), L and L_0_ are the final and initial lengths (m), respectively, g is the gravitational acceleration (9.81 m s^−2^), and RE is the resistance to extension (kg) recorded by the texture analyzer.

#### 2.4.5. Water Vapor Transmission Rate (WVTR) and Permeability (WVP)

WVTR and WVP of the nanofiber samples were determined at 25 °C and 40 °C under 90% relative humidity using a gravimetric method adapted from [35]. Falcon tubes (15 mL) containing dried silica gel (0% RH) were sealed with the nanofiber mats, providing an effective permeation area of 1.8 × 10^−4^ m^2^, and placed in a climate chamber (TK 252, Nuve, Ankara, Turkiye) at the corresponding temperature and 90% RH. Water vapor thus migrated from the humid environment through the nanofiber mat into the dry tube. Mass change was recorded at 15 time points over 24 h using an analytical balance (±0.0001 g), and the rate of mass change (ΔW/Δt) was obtained from the slope of the linear region of the mass–time curve. The water vapor transmission rate (WVTR) was calculated according to Equation (3):

(3) $$\mathrm{WVTR}=\left(\frac{\left(\frac{\Delta W}{\Delta t}\right)}{A}\right)$$

where ΔW is the mass change (g), Δt is the time interval (h), and A is the effective permeation area (m^2^). The ratio of water vapor transmission rate (R_WVTR_) was calculated according to Equation (4) to evaluate the thermoresponsive behavior of the nanofiber system:

(4) $$R_{\mathrm{WVTR}}=\left(\frac{{\mathrm{WVTR}}_{40{}^{\circ}C}}{{\mathrm{WVTR}}_{25{}^{\circ}C}}\right)$$

WVP was then determined according to Equation (5):

(5) $$\mathrm{WVP}=\left(\frac{\left(\mathrm{WVTR}\times x\right)}{∆P}\right)$$

where x is the sample thickness (Section 2.4.4) and ΔP is the water vapor partial pressure difference.

#### 2.4.6. GC-MS Analysis of the Nanofibers

The extractable amounts of the major REO markers in the nanofibers were determined by liquid-injection GC-MS. Briefly, 80 mg of nanofiber sample was dissolved in 10 mL of chloroform, and 2 µL was injected under the chromatographic conditions described in Section 2.2.2. α-Pinene, 1,8-cineole and camphor were quantified using external calibration curves prepared from the corresponding authentic standards over the range 0.25–6.25 µg mL^−1^ (R^2^ = 0.977, 0.997 and 0.999, respectively), and expressed as µg g^−1^ nanofiber. Borneol was tentatively identified by comparison with the Wiley library (SI = 92%) and semi-quantified using the camphor calibration curve; therefore, its content was expressed as camphor equivalents.

The release of volatiles from the nanofibers was evaluated by headspace solid-phase microextraction (HS-SPME) coupled to GC-MS according to the method described in [6]. Nanofiber samples (80 mg) were individually placed in 20 mL headspace vials under ambient laboratory conditions, hermetically sealed, and incubated at 25 or 40 °C for 4, 8, 12, 24 and 48 h, using a separate vial for each time point. Headspace volatiles were then extracted with a 75 µm Carboxen/PDMS SPME fiber for 30 min at the corresponding incubation temperature, thermally desorbed in the GC injector at 250 °C for 3 min. Chromatographic conditions were as described in Section 2.2.2. α-Pinene, 1,8-cineole and camphor were quantified against external calibration curves generated by HS-SPME at 25 °C over the ranges 1–25, 0.25–10 and 2.5–50 µg mL^−1^, respectively (R^2^ = 0.998, 0.980 and 0.998). Selected calibration points were additionally verified at 40 °C and showed comparable analytical responses. Borneol was semi-quantified as camphor equivalents as described above. The amount of each marker accumulated in the headspace was expressed as µg g^−1^ fiber and evaluated as a function of incubation time and temperature.

### 2.5. Antifungal and Antiaflatoxigenic Evaluation

#### 2.5.1. Selection of Fungal Strain

A total of 309 *Aspergillus* strains (304 *A. flavus* and 5 *A. parasiticus*) maintained as part of TUBITAK project (No. 119G001) were screened for aflatoxin production [37]. Following activation on PDA, spore suspensions were prepared in 0.05% Tween 80 and adjusted to 1 × 10^5^ spores mL^−1^ using a Thoma hemocytometer (Hawksley & Sons, Lancing, UK). Aliquots (100 µL) were inoculated onto Yeast Extract Sucrose (YES) agar and incubated at 26 ± 1 °C for 14 days. The *A. flavus* strain exhibiting the highest total aflatoxin production (3.9 × 10^4^ ng g^−1^) was selected for subsequent antifungal and antiaflatoxigenic assays.

#### 2.5.2. Aflatoxin Extraction and HPLC-FLD Analysis

Aflatoxins (AFB_1_, AFB_2_, AFG_1_, and AFG_2_) were extracted from the whole culture (agar and fungal biomass) with 100 mL of methanol/water (70:30, *v*/*v*) using a stomacher for 30 min, followed by filtration through a PTFE syringe filter [38]. Quantification was performed by HPLC-FLD (Shimadzu LC-20A, Kyoto, Japan) using a C18 column and a water/acetonitrile/methanol (6:2:3, *v*/*v*/*v*) mobile phase containing 119 mg L^−1^ KBr and 350 µL L^−1^ of 0.1 N HNO_3_. Separation was performed at 1.0 mL min^−1^ and 40 °C, with a fluorescence detection at 360/430 nm (Ex/Em) and a Kobra Cell current of 100 µA. Aflatoxins were quantified using external calibration (0.5–100 ppb), with coefficients of determination (R^2^) > 0.999 for AFB_1_, AFB_2_, AFG_1_, and AFG_2_, and results were expressed as ng g^−1^ of agar culture.

#### 2.5.3. Vapor-Phase Antifungal Activity of Nanofibers

Vapor-phase antifungal activity was assessed using a sealed Petri dish system (ISOLAB Chemicals, Eschau, Germany). PDA (20 mL) was dispensed into sterile Petri dishes to maintain a consistent headspace volume. Plates were centrally spot-inoculated with 1 μL of a fungal spore suspension (1 × 10^5^ spores mL^−1^), nanofiber samples were placed on the inner surface of Petri dish lid without direct contact with medium, sealed with parafilm, inverted, and incubated at 25 and 40 °C. To determine fiber amount for subsequent experiments, 15, 30 and 55 mg of PCL-L1 nanofibers were initially tested and 55 mg was selected. Subsequently, PCL-0, PCL-PN90, PCL-L1 and REO-PN90 were evaluated. Colony diameters were measured on days 2, 4, 7 and 10, and mycelial growth inhibition (MGI %) was calculated relative to the untreated control. Fungal cultures were harvested on day 7 at 25 °C and day 10 at 40 °C for subsequent aflatoxin analysis.

#### 2.5.4. Antiaflatoxigenic Activity of Nanofibers

Aflatoxins were extracted from nine agar plugs (1 cm diameter) excised from each fungal colony and transferred into glass tubes containing 20 mL of methanol/water (70:30, *v*/*v*) [39]. Samples were extracted under magnetic stirring at room temperature for 2 h, and aflatoxin levels were determined as described in Section 2.5.2. Concentrations are accordingly expressed as ng g^−1^ of agar culture.

### 2.6. Statistical Analysis

Statistical analysis was performed using SPSS software (IBM SPSS Statistics, Version 20). All experiments were performed in three independent replicates, and the results were expressed as mean ± standard deviation. Differences between groups were evaluated using one-way analysis of variance (ANOVA), followed by Tukey’s post hoc test. For the headspace volatile data of REO-loaded fibers, two-way ANOVA was performed to evaluate the effects of coating and temperature and their interaction, followed by Bonferroni-adjusted multiple comparisons. Differences were considered statistically significant at *p* < 0.05.

## 3. Results and Discussion

### 3.1. Volatile Composition of REO

GC-MS analysis identified six compounds accounting for approximately 87% of the total peak area (Table 4). The oil was dominated by oxygenated monoterpenes (~76%), mainly 1,8-cineole (46.51%) and camphor (21.09%), followed by borneol (7.86%), whereas monoterpene hydrocarbons (α-pinene, 8.67%; camphene, 2.42%) were present at lower proportions. Quantitative analysis of the selected marker compounds further showed that 1,8-cineole had the highest content (149.52 ± 0.71 µg mg^−1^ REO), followed by camphor (70.03 ± 0.05 µg mg^−1^ REO), α-pinene (52.63 ± 0.24 µg mg^−1^ REO), and borneol (25.76 ± 0.11 µg camphor equivalents mg^−1^ REO). Based on the relative peak areas, this distribution corresponds to the 1,8-cineole chemotype of *R. officinalis* [40] and falls within the wide compositional range previously reported for rosemary oils [41,42]. In contrast, an α-pinene-dominant profile has also been reported [29], in which α-pinene (20.67%) exceeded 1,8-cineole (9.38%).

### 3.2. Morphological Evaluation of Nanofibers (FESEM)

Representative FESEM micrographs and the corresponding fiber diameter distributions were presented in Figure 1. Continuous, randomly oriented and essentially bead-free fibers were obtained for all samples. Empty fibers (PCL-0) exhibited a mean diameter of approximately 950 nm, whereas REO incorporation (PCL-L1) significantly reduced the diameter to approximately 510 nm (*p* < 0.05). Similar reductions have been reported for EO-loaded PCL fibers and have been associated with EO-induced changes in the physicochemical properties of the spinning solution and consequent jet stretching during electrospinning [43,44]. However, the effect of EO incorporation on fiber diameter was system-dependent, and the opposite trends have also been reported [44]. Across the REO-loading series, PCL-L1 provided the most reproducible and continuous electrospinning and was therefore selected for subsequent characterization and PNIPAM coating.

PNIPAM modification resulted in an immersion-time-dependent increase in fiber diameter, from approximately 510 nm for the uncoated fibers to 650–830 nm after 30–120 s of immersion (Figure 1). The increase in diameter is consistent with progressive PNIPAM deposition during immersion in the reactive precursor mixture. Only a marginal increase was observed between 90 and 120 s (800 vs. 830 nm), suggesting limited additional deposition at longer immersion times. Similarly, a previous study [45] reported an increase in PCL fiber diameter following PNIPAM modification, from approximately 300–350 to 800–850 nm. The greater increase may be related to differences in the PNIPAM modification approach.

The elemental composition of the PCL-0 and PCL-PN90 fibers was compared by FESEM-EDS (Table 5). No nitrogen was detected in PCL-0, whereas a distinct nitrogen signal appeared in the PCL-PN90. Since nitrogen was absent from the PCL repeating unit, its presence after PNIPAM modification is attributable to the amide groups of PNIPAM gel, consistent with the emergence of a nitrogen signal following PNIPAM modification of PCL reported previously [46], and provides elemental evidence supporting its deposition on the fibers. The concurrent detection of the PCL-derived signals may reflect the EDS interaction volume extending beneath the PNIPAM-modified surface and including the underlying PCL. Oxygen, however, cannot serve as a specific indicator of PNIPAM, as it is present in both PCL and PNIPAM. Together with the FTIR results (Section 3.3), these findings further support PNIPAM modification of the fibers.

### 3.3. FTIR Analysis

The REO-free PCL nanofiber exhibited its characteristic absorption bands of PCL (Figure 2a), including carbonyl (C=O) stretching at ~1730–1710 cm^−1^, CH_2_ scissoring/bending at ~1470–1450 cm^−1^ and C-O-C stretching at ~1240–1260 cm^−1^ [47]. Upon REO incorporation, these characteristic bands remained essentially unchanged in wavenumbers, while the principal REO absorptions were largely masked by overlapping PCL bands. A weak shoulder at ~1330–1340 cm^−1^ was observed after REO incorporation and may be associated with C-H bending vibrations of the monoterpene constituents of REO [10].

PNIPAM modification and its dependence on immersion time were evaluated for the PCL-L1 fibers treated for 30–120 s (Figure 3a). The characteristic amide I (C=O stretching, ~1660–1630 cm^−1^) and amide II (N-H bending, ~1560–1530 cm^−1^) bands observed in the PNIPAM gel spectrum [48] became progressively more pronounced with increasing immersion time. This trend is consistent with increasing PNIPAM deposition within the ATR-probed region. The amide bands of REO-PN90 and REO-PN120 were comparable, suggesting limited additional deposition beyond 90 s, in agreement with the marginal increase in fiber diameter. Accordingly, 90 s was selected as the immersion time for subsequent analysis.

The spectrum of the selected PNIPAM-coated fiber (REO-PN90) was further compared with those of PNIPAM gel, REO, PCL-0 and PCL-L1 fibers (Figure 3b). The PNIPAM-associated amide I and amide II bands, absent in the uncoated fibers, were evident after modification, while the characteristic PCL bands and the REO-associated shoulder were retained. Together with the EDS results, these spectral features support the presence of PNIPAM in the modified fibers.

### 3.4. DSC Analysis

The melting endotherm of PCL polymer (61.73 °C) decreased to 59.30 °C after electrospinning, in line with previous reports attributing such changes to chain orientation and rapid solvent evaporation during fiber formation [49]. Incorporation of REO did not markedly affect the melting temperature (T_m_), which remained in a narrow range (58.80–60.56 °C) across REO-loaded fibers (Figure 2b).

DSC thermograms of the nanofibers and the PNIPAM gel are presented in Figure 3c. The PNIPAM gel exhibited a distinct endothermic transition at approximately 32–34 °C, corresponding to its lower critical solution temperature (LCST), at which the hydrated chains dehydrate and undergo the coil-to-globule transition [12]. This confirms the thermoresponsive character of the PNIPAM gel used in this study. The nanofiber samples similarly exhibited the characteristic PCL melting endotherm at approximately 58 °C; however, no distinct LCST-related endotherm was detected in the PNIPAM-coated fibers. The absence of a detectable LCST endotherm in the PNIPAM-coated fibers may be related to the sensitivity of DSC to the amount of thermoresponsive polymer present in the analyzed sample. Previous DSC measurements have shown that the heat associated with the LCST transition decreases markedly with decreasing PNIPAM concentration and may become undetectable at sufficiently low concentrations [34]. Considering that PNIPAM modification was independently supported by the FTIR and EDS results, the absence of a distinct LCST-related endotherm in the coated fibers should therefore not be interpreted as evidence for the absence of PNIPAM.

### 3.5. Contact Angle

The temperature-responsive wettability of the nanofibers was evaluated by dynamic water contact angle measurements below (25 °C) and above (40 °C) the LCST of PNIPAM gel (Figure 4). For the uncoated nanofibers, the contact angle showed no marked change over time at either temperature, reflecting their stable hydrophobic character. Notably, PCL-L1 showed a lower contact angle (96 ± 1.50°) than PCL-0 (115 ± 1.35°) at 25 °C (Figure 4a), which may reflect the combined effects of REO-induced morphological changes and the presence of oxygenated monoterpenes (e.g., 1,8-cineole and camphor), whose polar groups may enhance surface wettability. Comparable reductions in contact angle associated with altered fiber morphology and the presence of polar groups of the loaded compound have been reported for curcumin-loaded PCL and PLA fibers [50,51]. At 40 °C, however, the trend reversed, with PCL-L1 showing a higher contact angle (127 ± 0.92°) than PCL-0 (122 ± 1.26°). Since the water contact angle of polymeric surfaces generally shows only a weak dependence on temperature [52], this difference may instead indicate a temperature-induced change in surface composition. This may involve partial loss of the more volatile oxygenated constituents and/or redistribution of lipophilic REO components toward the fiber surface, thereby decreasing the apparent surface energy.

In contrast, the PNIPAM gel-coated nanofibers exhibited a pronounced temperature- and time-dependent wetting response. At 25 °C (<LCST), the droplets spread progressively over 10 s, with the contact angle decreasing from 115 ± 1.35° to 17 ± 1.96° for PCL-PN90 and from 62 ± 1.95° to 5 ± 1.35° for REO-PN90. At 40 °C (>LCST), droplet spreading was markedly suppressed with contact angles remaining at 44 ± 1.79° and 30 ± 1.79°, respectively, after 10 s (Figure 4b).

For PCL-PN90, the initial contact angles were comparable at both temperatures (~115°), indicating that the thermoresponsive behavior was expressed mainly through the wetting kinetics rather than static contact angle. This temperature-dependent wetting behavior may be associated with changes in PNIPAM hydration and conformation across the LCST. Below the LCST, hydrated PNIPAM chains interact favorably with water, whereas above the LCST, dehydration and conformational changes may reduce polymer-water interactions and suppress water spreading. However, complete chain collapse above the LCST should not be assumed, as the extent of PNIPAM conformational change can depend on the characteristics of the polymer system [53]. A comparable LCST-dependent wettability switch has been reported for PNIPAM gel-modified electrospun fibers [11]. Similarly, a temperature-dependent wettability switch has been reported for fibers coated by immersion and in situ radical polymerization, while the uncoated fibers were largely unaffected by temperature [26]. In their system, the transition was reflected in the static contact angle, whereas in the present study it was manifested primarily through changes in wetting kinetics. A temperature-dependent response was also observed in the water vapor transport measurements (Section 3.6), although these results were additionally influenced by mat thickness and the increased vapor-pressure driving force at 40 °C.

Interestingly, PNIPAM coating affected the initial wettability of the two fiber systems differently. At 25 °C, the initial contact angle remained essentially unchanged after PNIPAM-coating of REO-free PCL (115 ± 1.35°), whereas it decreased markedly from 96 ± 1.50° to 62 ± 1.95° for the REO-containing fibers. At 40 °C, PNIPAM coating decreased the initial contact angle of REO-free PCL from 122 ± 1.26° to 115 ± 1.53°, while a larger decrease from 127 ± 0.92° to 110 ± 1.82° was observed for the REO-containing fibers. Thus, at both temperatures, the effect of PNIPAM coating on the initial contact angle was more pronounced in the REO-containing system. The greater effect of PNIPAM coating on the REO-containing fibers may be partly related to their higher initial surface wettability, which could facilitate penetration of the aqueous coating solution into the fibrous network. However, since the difference between the uncoated fibers was not maintained at 40 °C, initial wettability alone cannot explain the coating response, and differences in PNIPAM distribution may also have contributed.

### 3.6. Water Vapor Transmission and Permeability

At 25 °C, PNIPAM-coating significantly reduced the WVTR of PCL-0 and PCL-L1 from 52.4 ± 0.74 and 52.6 ± 1.51 to 42.07 ± 1.88 (PCL-PN90) and 38.36 ± 1.11 g h^−1^ m^−2^ (REO-PN90), respectively (*p* < 0.05; Figure 5a). The lower WVTR values of the coated nanofibers may be largely associated with the greater thickness of the coated nanofibers, although changes in the fibrous structure following PNIPAM coating may also contribute to the observed WVTR [54,55]. The more pronounced reduction in WVTR observed for REO-PN90 may be related to differences in the surface wettability of the underlying fibers during the coating. REO incorporation decreased the water contact angle of PCL at 25 °C, which may have facilitated penetration of the aqueous PNIPAM coating solution and influenced its distribution within the fibrous network [45,46]. Accordingly, the slightly lower WVTR of REO-PN90 compared with PCL-PN90 may reflect differences in PNIPAM distribution, although the greater mat thickness should also be considered. Notably, REO incorporation alone did not affect WVTR at 25 °C, as PCL-L1 showed a value comparable to that of PCL-0. The larger decrease observed after PNIPAM coating therefore appears to be associated with the combined effects of coating and the resulting structural changes rather than a direct effect of REO on water vapor transmission.

At 40 °C, the differences in WVTR among the samples disappeared, with all samples showing similar values within a narrow range of 86.8–89.8 g h^−1^ m^−2^ (*p* > 0.05). Two-way ANOVA revealed a significant coating × temperature interaction for both REO-free and REO-containing fibers (*p* < 0.05), indicating that the effect of PNIPAM coating on WVTR depended on temperature. Bonferroni-adjusted pairwise comparisons showed that PNIPAM coating significantly reduced WVTR at 25 °C in both systems (*p* < 0.05), whereas no significant coating effect was observed at 40 °C (*p* > 0.05). Increasing the temperature from 25 to 40 °C significantly increased WVTR in both coated and uncoated fibers, regardless of REO incorporation (*p* < 0.05). This convergence of WVTR values at 40 °C may reflect the combined effects of the increased vapor-pressure driving force and thermoresponsive changes in PNIPAM hydration and conformation above its LCST [45]. To further assess the thermoresponsive behavior of the nanofibers, the water vapor transmission ratio (R_WVTR_ = WVTR_40_/WVTR_25_) was calculated (Figure 5a). According to Darcy’s law, the theoretical R_WVTR_ for a membrane operating between 25 and 40 °C at 90% RH is approximately 2.3 [54]. The uncoated PCL-0 and PCL-L1 nanofibers showed considerably lower ratios of 1.68 and 1.65, respectively, whereas the ratios increased to 2.13 for PCL-PN90 and 2.31 for REO-PN90. Thus, the higher R_WVTR_ values of the PNIPAM-coated fibers indicate a greater temperature dependence of WVTR, although this response should be interpreted together with the differences in mat thickness and WVP.

The water vapor permeability (WVP), which accounts for sample thickness and the water vapor pressure difference, was also evaluated (Figure 5b). At 25 °C, the coated PCL-PN90 and REO-PN90 showed higher WVP values (~ 0.50–0.52 g h^−1^ m^−1^ kPa^−1^) than their uncoated counterparts (0.37 g h^−1^ m^−1^ kPa^−1^). A similar pattern was observed at 40 °C, with the coated systems maintaining higher WVP values than the corresponding uncoated nanofibers. Two-way ANOVA revealed no significant coating × temperature interaction for WVP in either the REO-free or REO-containing fibers (*p* > 0.05). However, PNIPAM coating had a significant main effect in both systems (*p* < 0.05), whereas the main effect of temperature was not significant (*p* > 0.05). Bonferroni-adjusted pairwise comparisons showed that PNIPAM coating significantly increased WVP at both 25 and 40 °C, regardless of REO incorporation (*p* < 0.05), while the effect of temperature was not significant within either the coated or uncoated fibers (*p* > 0.05). Therefore, the lower WVTR of the coated nanofibers at 25 °C does not necessarily indicate an improved intrinsic water-vapor barrier, as the apparent reduction in transmission was influenced by the greater thickness of the coated mats. At 25 °C, the higher WVP of the coated systems may partly reflect the increased water affinity introduced by PNIPAM. Below its LCST (~32 °C), PNIPAM is hydrated and its amide groups interact favorably with water, which may increase water vapor sorption and facilitate transport through the coated structure, consistent with the increased moisture affinity of PNIPAM below its LCST [56].

### 3.7. Mechanical Properties of Nanofiber Systems

The tensile strength (TS) of the electrospun nanofibers was presented in Figure 6a. REO-free PCL-0 showed the lowest TS (1.23 ± 0.09 MPa), which increased to 1.62 ± 0.20 MPa after PNIPAM-coating. REO incorporation slightly increased the TS of the uncoated fibers to 1.38 ± 0.16 MPa, while subsequent PNIPAM coating further increased the TS to 1.62 ± 0.20 MPa. The low TS of PCL-0 may be associated with its loosely stacked fibrous network and limited fiber-to-fiber contacts, which play an important role in the mechanical strength of electrospun structures [57]. In contrast, the smaller and more uniform diameter of the REO-loaded fibers (~503 vs. ~941 nm) may have increased the density of contact points and promoted molecular orientation [58], partly accounting for their slightly higher, although non-significant, TS. This finding contrasts with reports in which essential oil incorporation lowered TS by disrupting fiber continuity [10]. A reinforcing effect of thermoresponsive polymer coating of fiber structures has previously been reported [59].

The elongation at break (EAB %) values of the samples were presented in Figure 6b. PCL-0 exhibited an EAB of 103.75%, which decreased to 93.68% following REO-incorporation and to 95.66% after PNIPAM coating. PNIPAM coating of the REO-loaded fibers resulted in no further change (93.99%). The lower EAB values of the modified fibers compared with PCL-0 were consistent with the slight increases in TS, suggesting a modest reduction in network extensibility, possibly due to restricted fiber mobility during tensile loading. Similar increases in TS accompanied by reduced extensibility have been reported for PNIPAM-coated fibrous substrates [59,60].

### 3.8. Extractable Marker Content and Temperature-Dependent Volatile Release Behavior of Nanofibers

Liquid-injection GC-MS of the dissolved fibers showed comparable marker contents in PCL-L1 and REO-PN90 (borneol 529.01 vs. 508.02, camphor 172.95 vs. 171.82, α-pinene 47.91 vs. 46.48, and 1,8-cineole 44.77 vs. 40.95 µg g^−1^ fiber; *p* > 0.05) **(**Figure 7a). These results indicate that the PNIPAM coating did not cause appreciable loss of REO volatiles from the fibers. Interestingly, the marker distribution within the fibers differed markedly from that of the original REO, with borneol and camphor becoming predominant, whereas α-pinene and 1,8-cineole were present at considerably lower levels. This shift broadly follows the volatility of the compounds, with boiling points decreasing in the order borneol (~213 °C) > camphor (~204 °C) > 1,8-cineole (~176 °C) > α-pinene (~156 °C). Preferential loss of more volatile essential-oil constituents during electrospinning has previously been reported [61], suggesting that differences in volatility may have contributed to the altered marker profile observed in the fibers.

Headspace SPME analysis showed that each marker accumulated over time, with consistently higher amounts at 40 °C than at 25 °C (Figure 7b–e), consistent with temperature-enhanced volatilization and headspace partitioning of monoterpenes [62]. Headspace amounts were substantially lower than extractable marker contents because the latter represent compounds recovered after fiber dissolution, whereas HS-SPME measures only the gas-phase fraction sampled during the 30 min extraction. Even after 48 h at 40 °C, headspace amounts corresponded to less than approximately 2% of the extractable marker contents.

Coating- and temperature-dependent differences were compared at 48 h, the final sampling point. After 48 h, the four markers together accounted for 3.773 ± 0.51 and 6.229 ± 0.38 µg g^−1^ fiber in PCL-L1 at 25 and 40 °C, respectively, compared with 0.519 ± 0.03 and 1.252 ± 0.03 µg g^−1^ fiber in REO-PN90 (Figure 7b–e). Although headspace accumulation increased numerically with temperature in both systems, the magnitude and statistical significance of this change differed between the coated and uncoated fibers. PNIPAM coating markedly reduced headspace accumulation at both temperatures, by approximately 7.2-fold at 25 °C and 4.9-fold at 40 °C. Since the extractable marker contents were comparable (Figure 7a), this reduction suggests that the PNIPAM coating restricted the transfer of volatiles from the fibers to the headspace, rather than reducing their amount in the fibers during immersion-based preparation method. Two-way ANOVA revealed a significant coating × temperature interaction (*p* < 0.05), indicating that the effect of temperature on headspace volatile accumulation depended on the presence of the PNIPAM coating. Bonferroni-adjusted pairwise comparisons further showed that increasing the temperature from 25 to 40 °C significantly increased headspace accumulation in PCL-L1 (*p* < 0.05), whereas the increase observed in REO-PN90 did not reach statistical significance (*p* = 0.085). The temperature-dependent modulation observed in the coated fibers was more moderate than that reported for systems containing a thick and continuous PNIPAM layer. This difference may partly arise from the coating geometry. In the present study, immersion coating did not form a distinct continuous layer over the fibrous mat but was mainly reflected by an increase in fiber diameter (Figure 1). A dependence of release behavior on PNIPAM coating thickness has been reported for PLA/PNIPAM core–shell fibers, where increasing shell thickness progressively slowed release [11]. The release environment may also have contributed to the observed behavior. Unlike studies conducted in aqueous media, the present headspace experiments were performed in sealed vials without liquid medium, representing a vapor-phase environment more relevant to food-packaging applications. The behavior of PNIPAM under such conditions may differ from that observed during immersion in water, which could partly account for the relatively moderate temperature-dependent modulation of volatile transfer observed here.

### 3.9. Inhibition of Aspergillus Flavus Growth

The vapor-phase antifungal activity of the coated and uncoated nanofibers against *A. flavus* was followed over the incubation period, and the MGI values were summarized in Table 6. Since the fibers were fixed to the lid of the inverted plates, the fungus was exposed to volatiles transferred from 55 mg of fiber into the sealed headspace, without direct contact with the material. The PCL-0 and PCL-PN90 nanofibers caused only marginal inhibition, not exceeding 9% on day 2 and remaining below 2% thereafter, indicating that the antifungal activity of the REO-loaded systems was primarily associated with REO volatiles rather than with the PCL matrix or PNIPAM coating. REO, containing several of the monoterpenes identified in the present study, has previously been reported to inhibit the mycelial growth of *A. flavus* [5]. However, the four markers quantified by HS-SPME represent only part of the volatile profile of REO, which also contained camphene, linalool and unidentified constituents. The observed inhibition should therefore be considered as the combined effect of the volatile compounds transferred to the headspace rather than any individual marker. At 25 °C, PCL-L1 and REO-PN90 inhibited fungal growth to a comparable extent on day 2 (19.30 and 15.79%, *p* > 0.05; Table 6), but the effect declined sharply thereafter. By day 4, only the uncoated fibers showed significantly greater inhibition than the REO-free controls, while by day 7 neither formulation was distinguishable from corresponding REO-free controls. The observed behavior suggests that the volatile levels established in the headspace were sufficient to delay early fungal development but not to sustain growth inhibition over prolonged incubation. Such a transient effect is consistent with the extended lag phase reported for *A. flavus* exposed to essential oil vapors [63].

At 40 °C the inhibitory effect was sustained throughout the incubation period. PCL-L1 maintained an MGI of 22.5–27.4% up to day 10 and remained the most active formulation at every sampling point (*p* < 0.05; Table 6), an effect also evident in the plate images (Figure 8). REO-PN90 also maintained measurable inhibition (7.6–17.3%), which was significantly higher than that of the REO-free samples from day 4 onwards. However, REO-PN90 consistently showed lower inhibition than the uncoated REO-loaded fibers (PCL-L1). The greater persistence of antifungal activity at 40 °C may be associated with two factors that cannot be distinguished in the present experimental design. First, the higher temperature increased the accumulation of REO volatiles in the headspace, as demonstrated by the HS-SPME results. Second, 40 °C is above the temperature range supporting the most rapid growth of *A. flavus*, consistent with the slower development of the control colony, which required approximately 10 days to cover the plate compared with 7 days at 25 °C [15]. Importantly, this temperature-dependent pattern was observed for both coated and uncoated REO-loaded fibers and therefore cannot be attributed specifically to the PNIPAM transition.

### 3.10. Effect on Aflatoxin Production

Aflatoxin accumulation was determined at the end of each incubation (day 7 at 25 °C and day 10 at 40 °C; Table 6). Only AFB_1_ was detected; AFB_2_, AFG_1_ and AFG_2_ remained below the limit of detection (0.01 ng g^−1^) in all samples, in agreement with the profile of the selected isolate and with the initial screening, in which none of the strains produced AFG_1_ or AFG_2_. At 25 °C, the untreated control accumulated 14.24 ng g^−1^ AFB_1_, and none of the nanofiber systems, including the REO-loaded formulations, significantly affected AFB_1_ accumulation (*p* > 0.05). Thus, although the REO-loaded fibers transiently inhibited fungal growth during the early stages of incubation, this effect was not accompanied by a significant reduction in AFB_1_ accumulation at the end of the 7-day incubation.

Growth inhibition and suppression of aflatoxin biosynthesis do not necessarily occur in parallel; differential effects of essential oils on these two responses have previously been reported for *A. flavus* [64]. At 40 °C, AFB_1_ was not detected in any sample, including the untreated control, preventing assessment of the antiaflatoxigenic effect of the nanofiber systems. Aflatoxin production at elevated temperatures is strongly dependent on strain and environmental conditions. AFB_1_ has been reported only between 25 and 35 °C in some *A. flavus* isolates [15]. However, under high water activity, a high-producing isolate still accumulated AFB_1_ at 42 °C, although at considerably lower levels than at 34 °C, whereas a low-producing isolate did not [65].

The 40 °C condition was selected to ensure that the PNIPAM coating was evaluated above its transition temperature rather than to reproduce conditions optimal for aflatoxin production. Future studies could therefore evaluate the antiaflatoxigenic performance of thermoresponsive systems at temperatures closer to this transition, under conditions that support measurable AFB_1_ production.

## 4. Conclusions

In this study, REO was incorporated into electrospun PCL nanofibers, which were subsequently modified with a thermoresponsive PNIPAM-gel coating using an immersion-based approach. The presence of PNIPAM was supported by EDS and FTIR analyses, and the coated fibers exhibited clear temperature-dependent surface wetting behavior below and above the LCST. For REO-PN90, the contact angle decreased from approximately 62° to 5° within 10 s at 25 °C, whereas it remained at approximately 30° after 10 s at 40 °C, demonstrating a pronounced temperature-dependent difference in wetting behavior. PNIPAM coating also increased the WVTR_40_/WVTR_25_ ratio of the REO-loaded fibers from 1.65 to 2.31, indicating greater temperature dependence of water vapor transmission, although the thickness-normalized WVP did not indicate a distinct LCST-dependent permeability switch.

Headspace analysis demonstrated that PNIPAM coating substantially restricted the transfer of REO volatiles to the gas phase at both 25 and 40 °C. After 48 h, PNIPAM coating reduced the total headspace amount of the selected volatile markers by approximately 7.2-fold at 25 °C and 4.9-fold at 40 °C. The significant coating × temperature interaction indicated that the coating also modified the temperature dependence of volatile transfer, with a greater relative increase in headspace accumulation from 25 to 40 °C for the coated fibers. Thus, PNIPAM coating primarily promoted volatile retention while providing temperature-dependent modulation rather than a simple LCST-triggered release response.

REO-loaded fibers showed vapor-phase antifungal activity against *A. flavus*, with transient inhibition at 25 °C and more persistent inhibition at 40 °C. The lower antifungal activity of the PNIPAM-coated fibers compared with the uncoated REO-loaded fibers at 40 °C was consistent with their lower transfer of volatiles to the headspace. In contrast, none of the systems significantly affected AFB_1_ accumulation at 25 °C, while AFB_1_ was not detected in any sample, including the untreated control, at 40 °C, preventing evaluation of an antiaflatoxigenic effect above the LCST.

Our findings demonstrate that PNIPAM surface modification can substantially enhance the retention of EO volatiles during incubation and modulate temperature-dependent of volatile transfer. Future studies should extend headspace monitoring beyond 48 h and evaluate volatile transfer and antifungal or antibacterial performance under controlled high-relative-humidity conditions that favor growth. Such conditions would also allow further investigation of whether PNIPAM hydration contributes to the observed temperature-dependent modulation of volatile transfer.

## Figures and Tables

**Figure 1:**
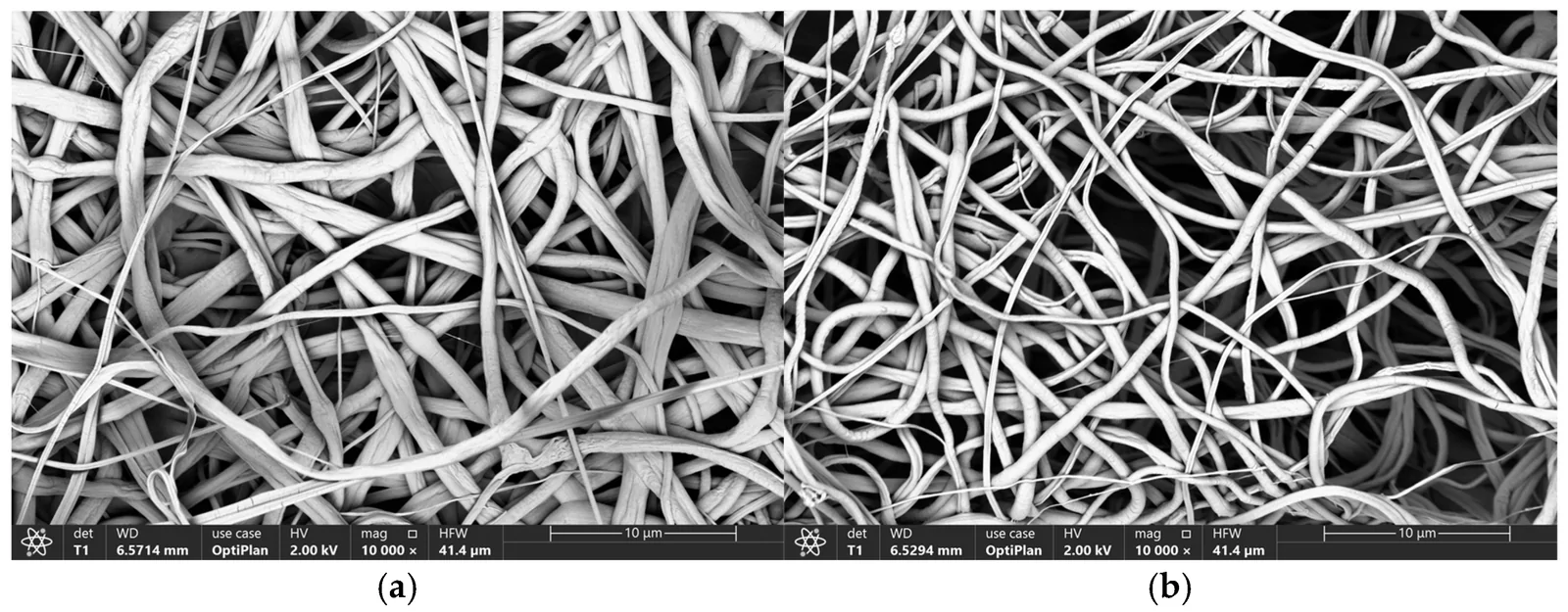

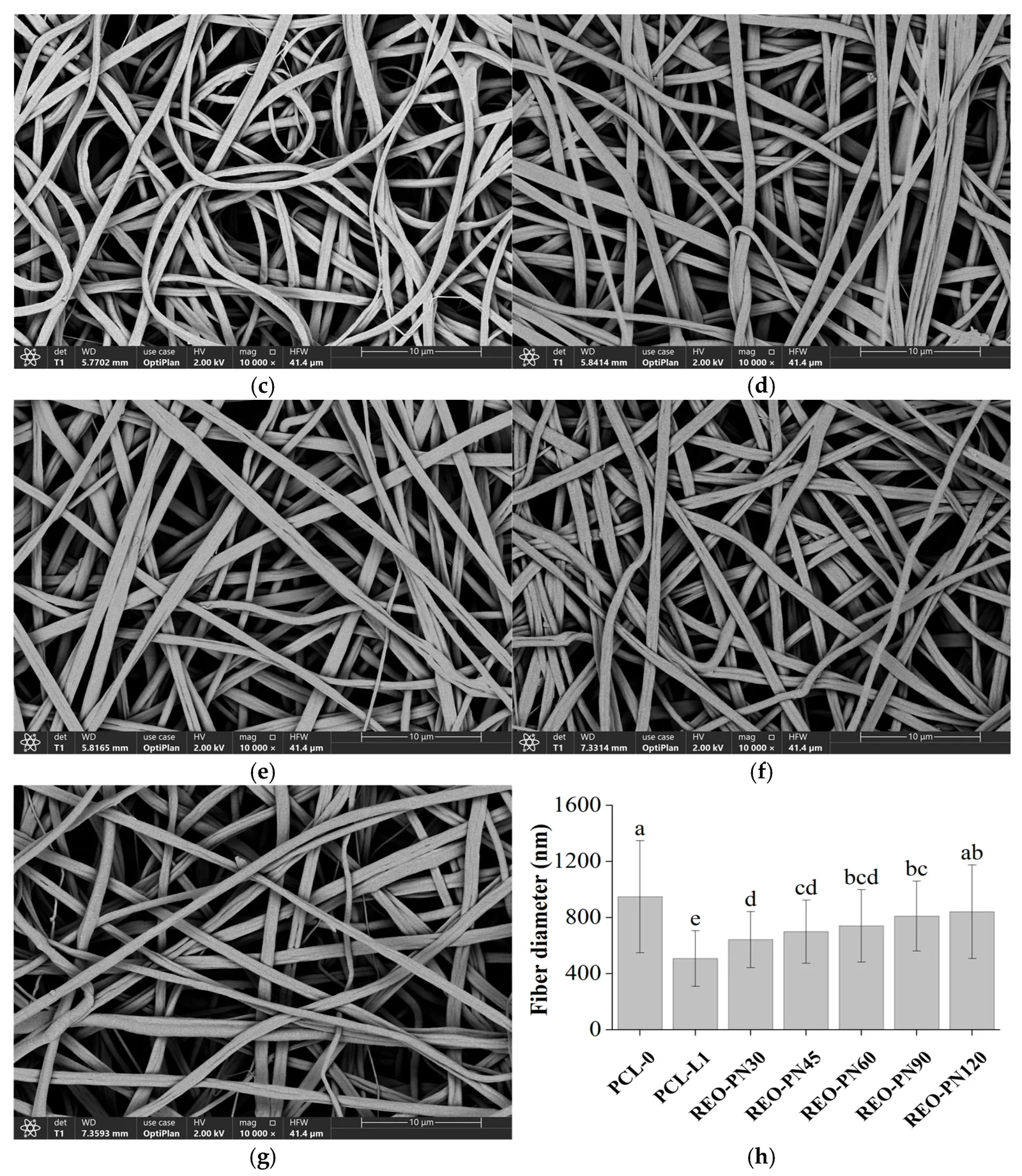
Effect of PNIPAM gel immersion time on the morphology of REO-loaded electrospun nanofibers. (**a**) PCL-0, (**b**) PCL-L1, (**c**) REO-PN30, (**d**) REO-PN45, (**e**) REO-PN60, (**f**) REO-PN90, and (**g**) REO-PN120. (**h**) Average fiber diameter of the corresponding samples (*n* = 100). Different letters indicate statistically significant differences among the samples (*p* < 0.05). Scale bar = 10 μm.

**Figure 2:**
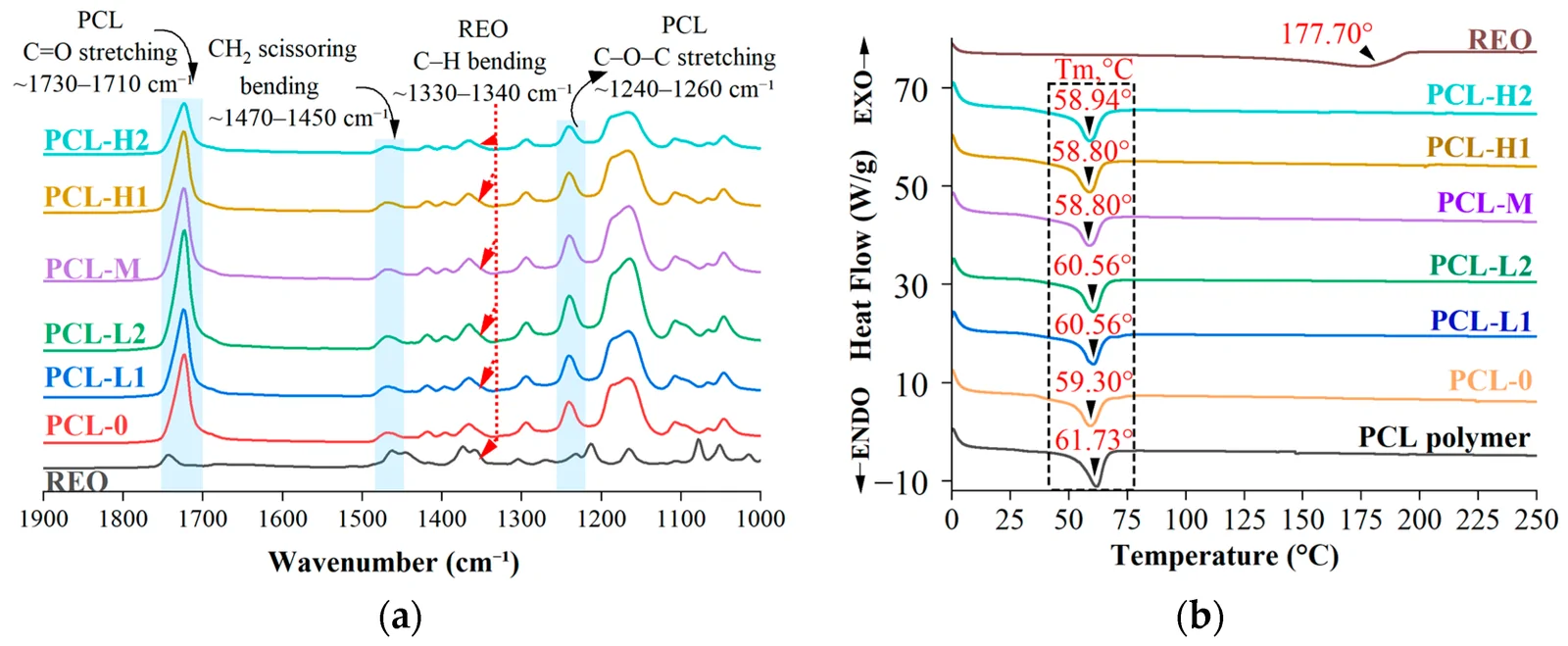
(**a**) FTIR spectra and (**b**) DSC thermograms of PCL polymer, PCL nanofibers, REO and REO-loaded formulations with varying REO contents.

**Figure 3:**
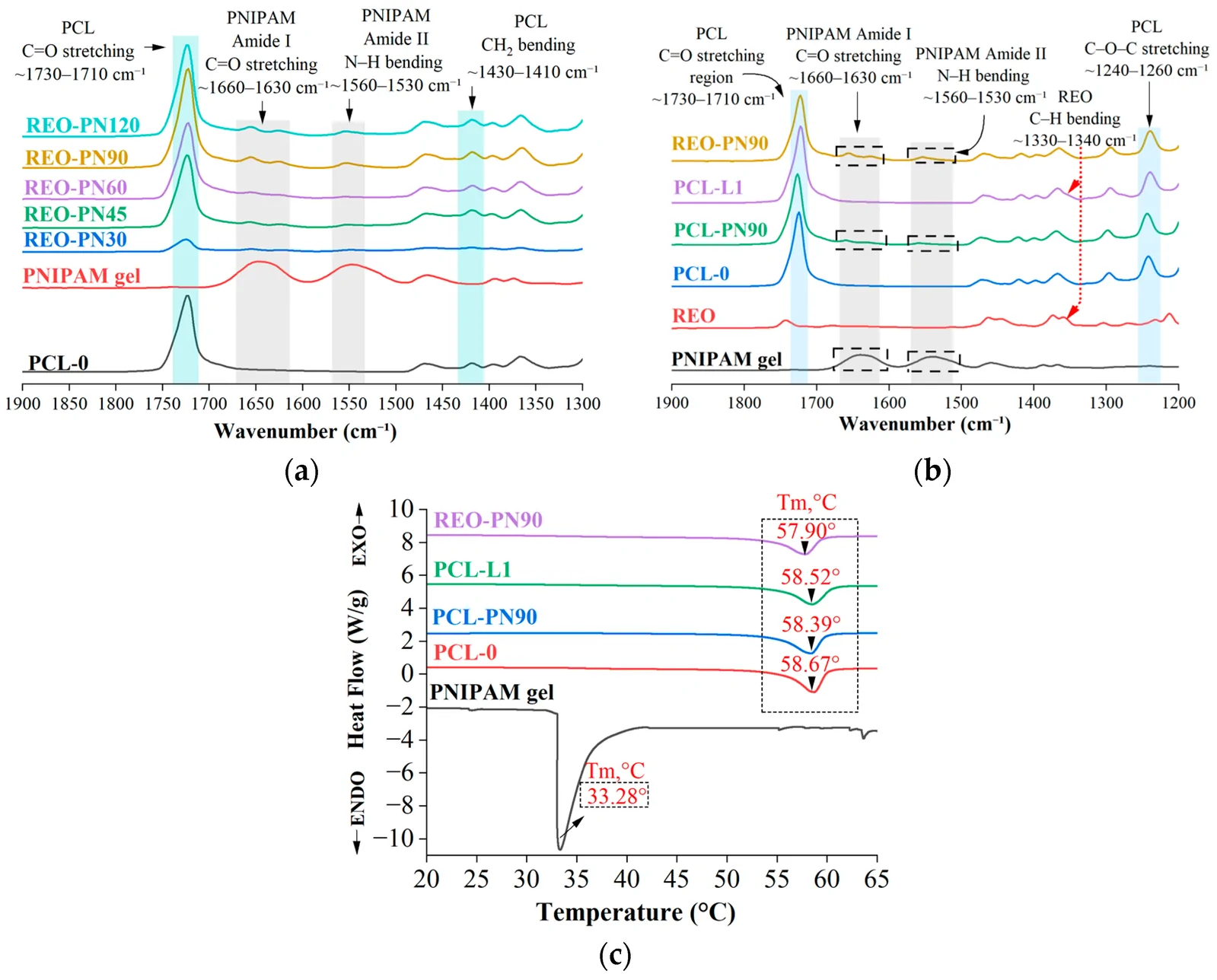
(**a**) FTIR spectra of PCL-L1 nanofibers at different immersion times, (**b**) FTIR spectra of PNIPAM gel, REO, and nanofiber formulations, and (**c**) DSC thermograms of PNIPAM gel and nanofiber formulations.

**Figure 4:**
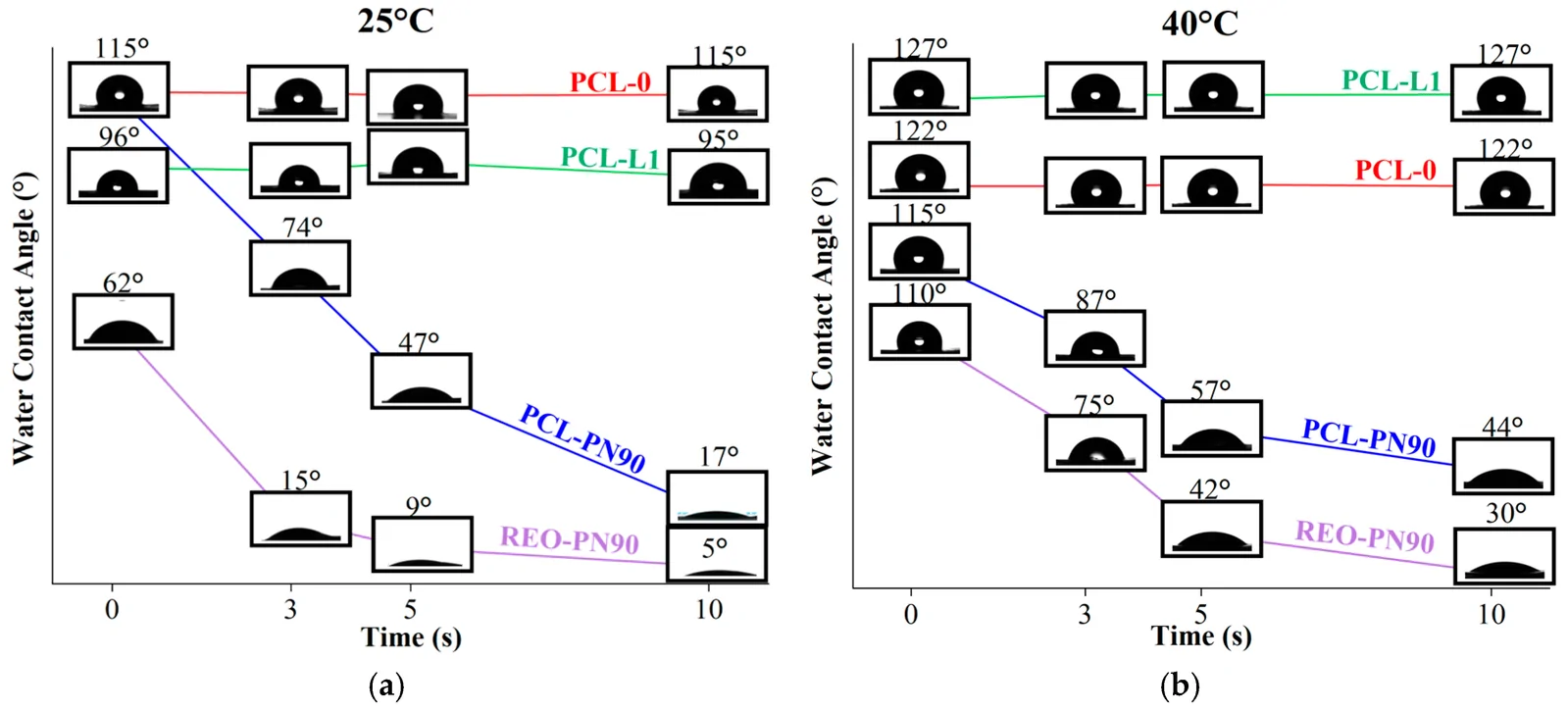
Dynamic water contact angles of the PCL and PNIPAM gel-coated nanofibers over 10 s at (**a**) 25 °C, below the LCST, and (**b**) 40 °C, above the LCST of PNIPAM.

**Figure 5:**
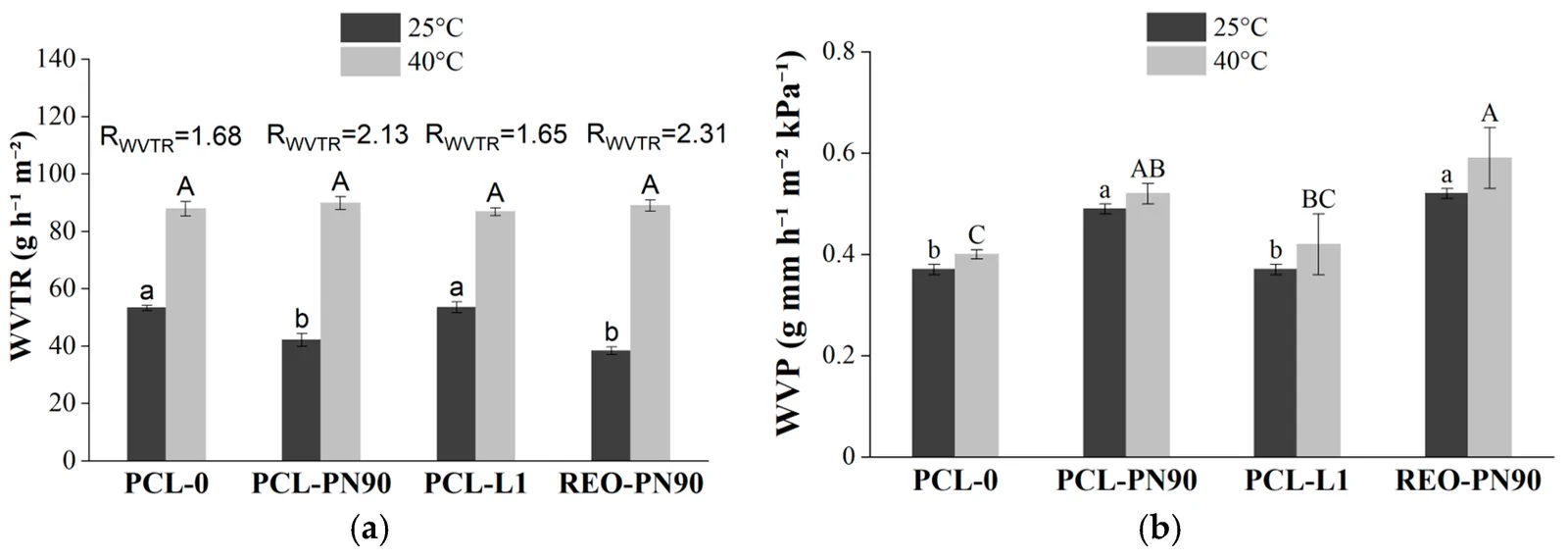
Temperature-dependent (**a**) water vapor transmission rate (WVTR) and the corresponding temperature response ratio (Rwvtr) at 25 and 40 °C and (**b**) water vapor permeability (WVP) of nanofiber systems. Different lowercase and uppercase letters indicate statistically significant differences among samples at 25 and 40 °C, respectively (*p* < 0.05).

**Figure 6:**
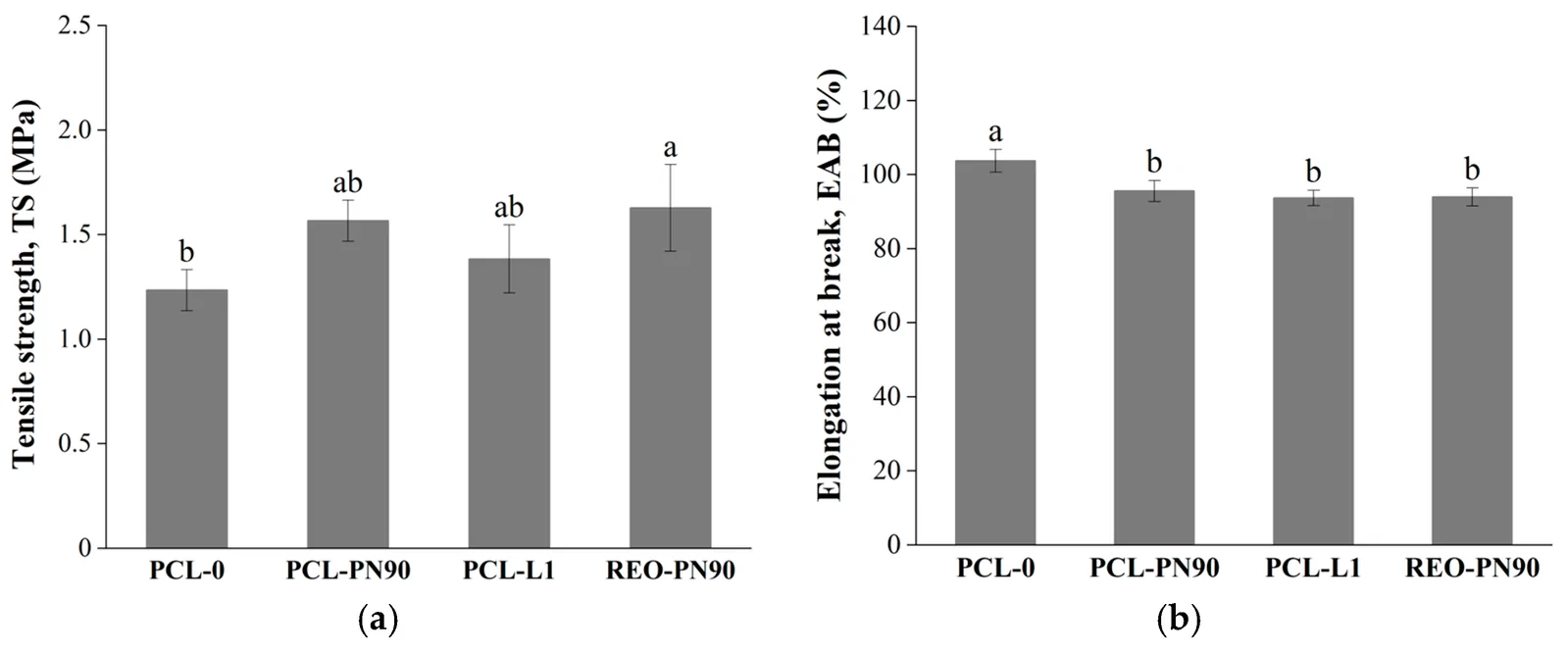
Mechanical properties: (**a**) tensile strength (TS) and (**b**) elongation at break (EAB) of PCL-0, PCL-PN90, PCL-L1 and REO-PN90 nanofibers. Different lowercase letters indicate statistically significant differences among samples for the same parameter (*p* < 0.05).

**Figure 7:**
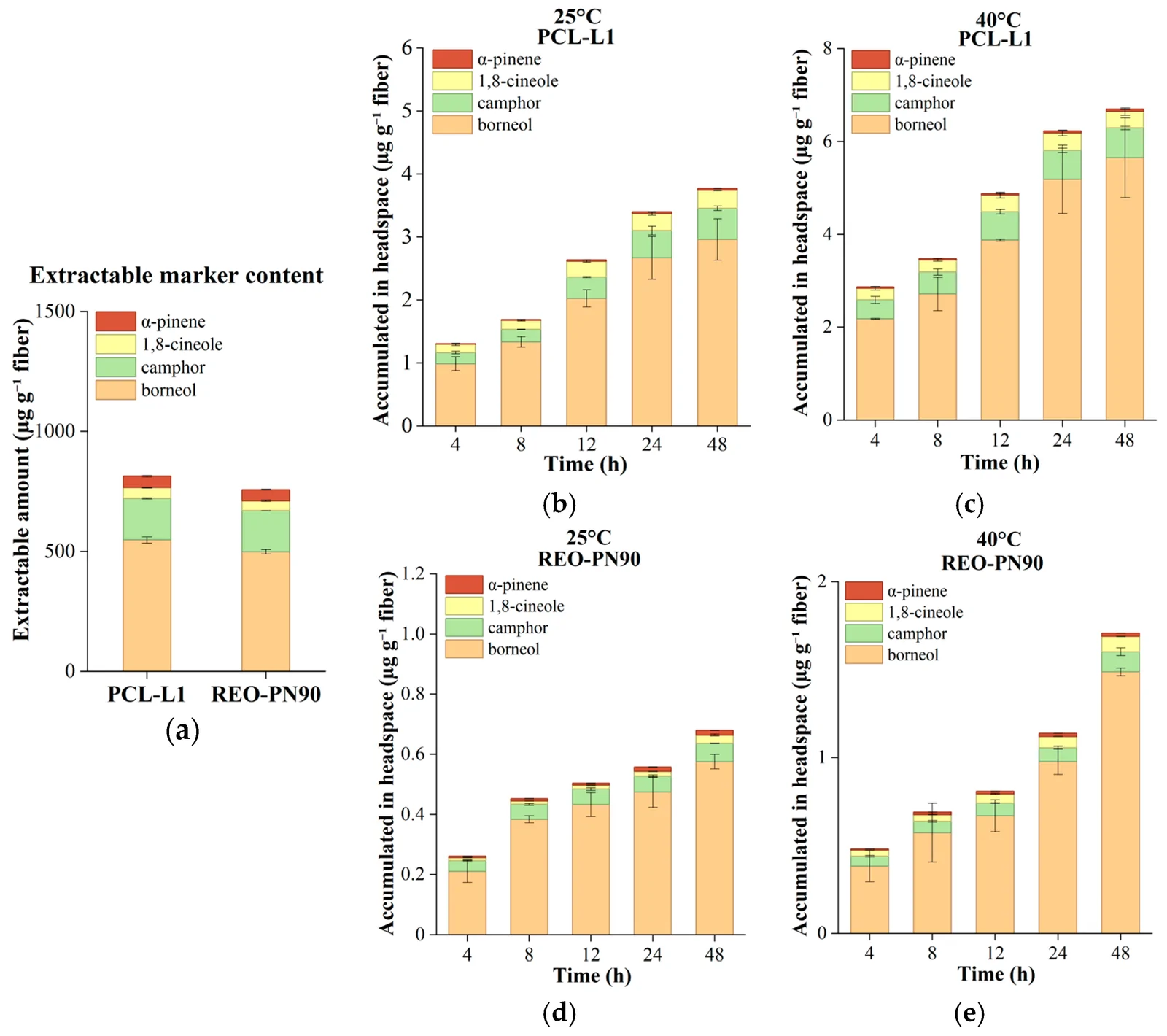
Extractable content and headspace accumulation of four major REO markers (α-pinene, camphor, 1,8-cineole, and borneol) in PCL-L1 and REO-PN90 nanofibers. (**a**) Extractable marker content determined by liquid-injection GC-MS. (**b**–**e**) Headspace accumulation measured by HS-SPME-GC-MS after 4, 8, 12, 24, and 48 h for PCL-L1 at 25 °C (**b**) and 40 °C (**c**), and REO-PN90 at 25 °C (**d**) and 40 °C (**e**). Borneol was semi-quantified as camphor equivalents.

**Figure 8:**
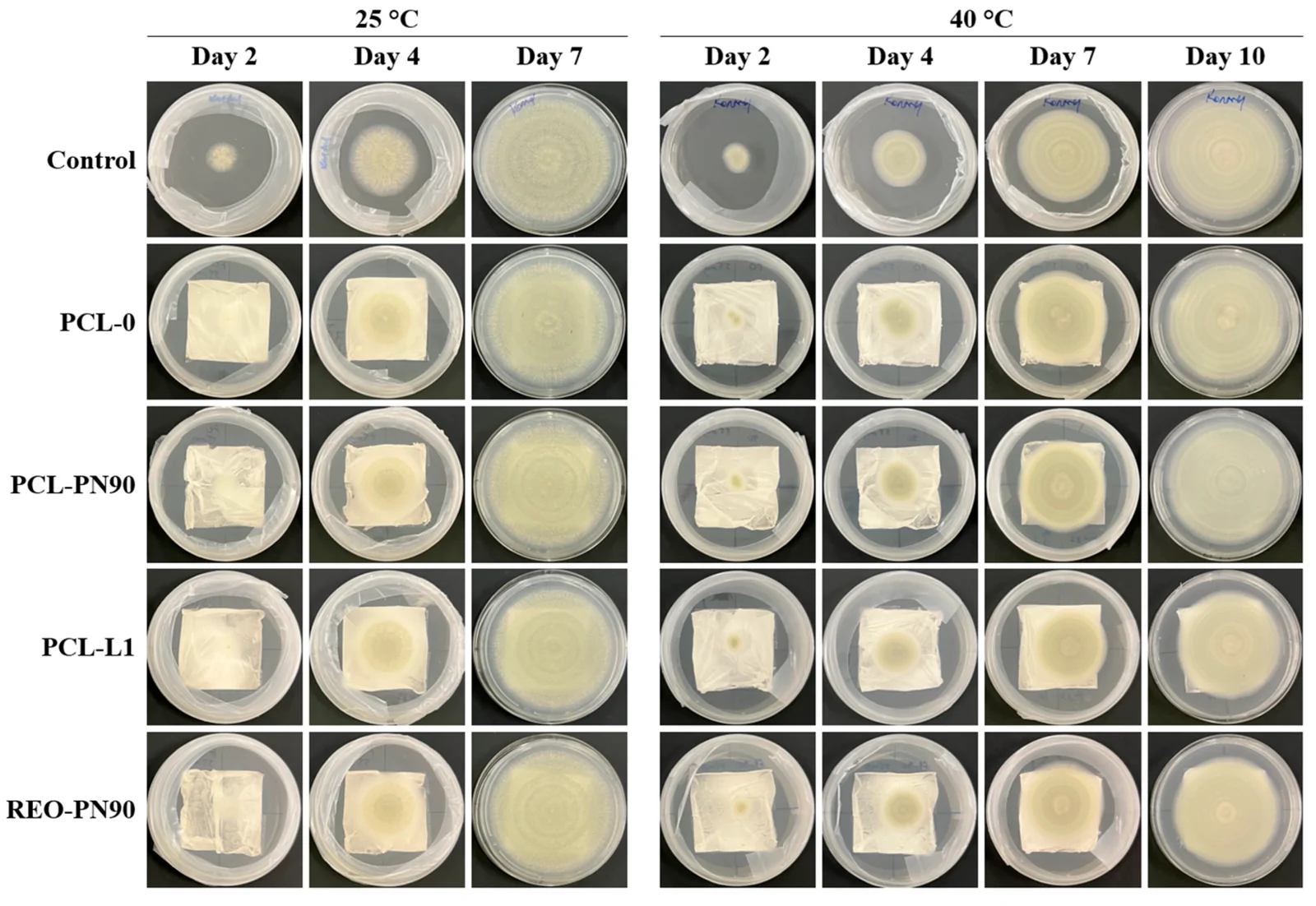
Visual comparison of *Aspergillus flavus* growth under vapor-phase exposure to PCL-based nanofiber systems at 25 and 40 °C.

**Table 1:** The summary of previous studies on thermoresponsive polymer-based systems developed for the controlled release of bioactive and antimicrobial compounds in food and biomedical applications.

System Architecture	Fabrication Method	Thermoresponsive Element & Transition	Application & Potential Target	Key Outcomes	Ref.
Poly(vinyl alcohol) (PVA)/poly(*N*-vinylcaprolactam) (PNVCL) shell/-poly(lactic acid) (PLA)/CEO core nanofibrous film	Coaxial electrospinning; PLA/CEO Cinnamon EO core and PVA/PNVCL shell, forming a sandwich-like fibrous structure	PNVCL in the PVA/PNVCL shell; LCST ~32–35 °C	Postharvest preservation of Chinese bayberry	CEO release ↓ above LCST; mechanical performance ↑; weight loss and softening ↓; fruit quality retention ↑; antibacterial (*E. coli*, *S. aureus*) and antifungal (*A. niger*) activity ↑	[13]
PNIPAAM/PVA-PLA/LEO (Lemon EO) nanofibrous films with uniaxial, core–shell and layered architectures	Uniaxial/blend, coaxial core–shell and layered sandwich electrospinning	PNIPAAM; LCST~32 °C	Postharvest preservation of blackberries	LEO release ↓ below and ↑ above LCST; release control ↑ in coaxial/layered films; antibacterial (*E. coli*, *S. aureus*) and antifungal (*A. japonicus*, *G. persicaria*) activity ↑; blackberry decay and softening ↓	[14]
PPC/CEO electrospun fibrous mat coated with a conductive PEDOT:PSS interlayer and a PNIPAM top layer	Electrospinning; dip-coating (PEDOT:PSS, 30 s); spin-coating of UV-polymerised PNIPAM	PNIPAM; LCST~32 °C	Voltage-triggered on-demand antioxidant release for active food packaging	CEO release ↓ below LCST (~4%) and ↑ above LCST (~64%) at 2 V; DPPH ↑ (15→95%); contact angle ↑ (27→64°); degradation temp. ↑ (241→297 °C, final device)	[19]
PNIPA/AA (acrylic acid) nanohydrogels incorporated into κ-carrageenan/locust bean gum polysaccharide films	Water-in-oil microemulsion polymerization + solution casting (Natamycin)	PNIPA/AA; LCST~33 °C	Controlled natamycin release for active food packaging	Natamycin release ↑ above LCST; lag time ↑ (~35 h); sustained release ↑ with PNIPA-co-AA; natamycin stability ↑; anti-yeast activity against *S. cerevisiae* ↑	[20]
PNIPA-20AA(5) nanohydrogel coating	Microemulsion polymerization and coating application (Natamycin)	PNIPA-AA; LCST~33 °C	Arzúa-Ulloa DOP cheese preservation	Controlled natamycin release ↑; total fungal counts ↓; antifungal protection ↑; cheese ripening ↔	[21]
PNIPA-20AA(5) nanohydrogel	Water-in-oil microemulsion polymerization; pimaricin loading (Natamycin)	PNIPA-AA; LCST~33 °C	Grape juice (yeast control)	Controlled natamycin release ↑; *S. cerevisiae* inhibition ↑; inhibition duration ↑; natamycin stability ↑	[22]
Thymol-loaded PNIPAM/ Poly(*N*-*tert*-butylacrylamide)(PBAM)/poly(acrylic acid) (PAA) nanocomposite hydrogel adsorbed on chitosan film	In situ polymerization; hydrogel adsorbed onto cast film	PNIPAM/PBAM/PAA; temperature-, pH- and salt-responsive (LCST depending on PBAM ratio)	Active antibacterial food packaging film	Thymol release rate ↑ above LCST; sustained thymol release ↑ below LCST; antibacterial activity against *B. subtilis* and *E. coli* ↑	[23]
AgNP-loaded PDA@PNIPAM/basalt epoxy composite coating (APEB/EP)	PDA self-polymerization; PNIPAM shell polymerization; AgNP deposition; spray coating	PNIPAM; LCST~32 °C	Anti-corrosion antibacterial coating	Ag^+^ release ↑ below LCST; bacterial adhesion ↓ above LCST; adhered bacteria removal ↑ below LCST; long-term antibacterial and anticorrosion performance ↑	[24]
BSA (bovine serum albumin)-conjugated core–sheath PCL/PNIPAAM nanofibers	BSA conjugation; single-spinneret electrospinning; UV thiol–ene crosslinking	PNIPAAM sheath; LCST~32 °C	Erythrocyte (RBC) capture and release	RBC capture ↑ above LCST; RBC release ↑ below LCST; platelet adhesion ↓; capture/release efficiency ↑ (up to 100%);RBC integrity ↔	[25]
Combretastatin (CA4)-loaded PLA core/PNIPAM shell fibers	Electrospinning; UV-induced PNIPAM shell polymerization	PNIPAM shell; LCST~32 °C	Anticancer drug delivery	CA4 release ↑ above LCST; sustained release ↓ below LCST; drug release ↓ with increasing PNIPAM shell thickness; low cytotoxicity and good biocompatibility	[11]
Curcumin-loaded PES core/P(NIPAm-co-Am) shell ultrafine fibers	Electrospinning; radical copolymerization of P(NIPAm-co-Am) shell	P(NIPAM-co-Am); LCST raised via acrylamide (~43 °C)	Hyperthermia-associated drug delivery	Curcumin release ↑ above LCST; thermoresponsive pore opening ↑ drug release; good biocompatibility	[26]
ZnO-loaded PNIPAM/PVA semi-IPN nanofibrous mats	Solution radical polymerization; electrospinning; glutaraldehyde vapor crosslinking	PNIPAM; LCST ~32 °C	Wound dressing	ZnO release ↓ above LCST; thermoresponsive antibacterial activity against *S. aureus*; good cytocompatibility; enhanced wound healing	[17]
PHT (Phenytoin)-loaded poly(lactic-*co*-glycolic acid) (PLGA) nanoparticle-embedded GelMA/NIPAM hydrogels	Single emulsion-solvent evaporation; UV photopolymerization; nanoparticle embedding	PNIPAM; LCST ~32 °C	Antiepileptic drug delivery	PHT release ↑ at 40 °C; sustained drug release; reversible swelling–shrinking; good cytocompatibility	[27]

The symbols used in the key outcomes column indicate the direction of the reported effect: ↑ denotes an increase, ↓ a decrease and ↔ no change in the corresponding parameter, either with respect to the control formulation or to the value determined below the transition temperature, as reported in the cited study.

**Table 2:** The formulation matrix and electrospinning parameters of REO-loaded nanofibers.

Sample Code	PCL:REO Ratio (*w w*^−1^)	PCL Concentration (% *w v*^−1^)	CHCl_3_:DMF (*v v*^−1^)	Distance (cm)	Voltage (kV)	Flow Rate (mL h^−1^)
PCL-0	1:0	15	3:2	11	17	0.2
PCL-L1	21.37:1	15	3:2	11	17	0.2
PCL-L2	9.92:1	15	4:1	9	14	0.2
PCL-M	2.11:1	10	4:1	11	14.5	0.1
PCL-H1	1:1	10	4:1	13	18.5	0.1
PCL-H2	1:2.25	10	4:1	13	18.5	0.1

CHCl_3_:DMF: chloroform: N,N-dimethylformamide; L, M and H indicate low, medium, and high REO loading, respectively. PCL/REO ratios (*w w*^−1^) were calculated using the density of REO.

**Table 3:** Sample nomenclature of PNIPAM-coated nanofibers.

Category	Code	PNIPAM Immersion Time (s)
REO-free control	PCL-0	0
	PCL-PN90	90
REO-loaded fibers	PCL-L1	0
	REO-PN30	30
	REO-PN45	45
	REO-PN60	60
	REO-PN90	90
	REO-PN120	120

PN denotes PNIPAM gel coating, and the number indicates the immersion time (s) in the precursor solution; PCL-L1 represents the uncoated REO-loaded fiber.

**Table 4:** Chemical composition of rosemary essential oil (REO) determined by GC-MS.

Peak Number	RT (min)	Component	%Area	Main Ions (*m*/*z*)	%SI
1	9.58	α-pinene	8.67	93, 121, 136	97
2	10.02	camphene	2.42	93, 121, 136	92
3	12.39	1,8-cineole	46.51	81, 108, 154	98
4	14.23	linalool	0.47	71, 93, 121	90
5	15.36	camphor	21.09	81, 95, 108	92
6	15.94	borneol	7.86	95, 110, 139	92
	-	Unidentified compounds	12.98	-	<90
∑ Peak Area %	-		100	-	-

Compound identification was performed by comparison of mass spectra with the Wiley mass spectral library. RT: retention time; SI: similarity index. Compounds with a similarity index (SI) below 90 were classified as unidentified.

**Table 5:** EDS elemental composition (weight %) of PCL and PCL-PN90 nanofibers.

	C	N	O
	wt%		
PCL-0	56.6 ± 0.2	n.d.	43.4 ± 0.2
PCL-PN90	60.9 ± 0.3	5.1 ± 0.5	34 ± 0.3

n.d. = not detected.

**Table 6:** Vapor-phase antifungal activity (mycelial growth inhibition, MGI%) and aflatoxin B_1_ accumulation of *A. flavus* exposed to PCL-based nanofiber systems at 25 and 40 °C.

	25 °C MGI (%)				40 °C MGI (%)				
Sample	Day 2	Day 4	Day 7	AFB_1_ (ng g^−1^)	Day 2	Day 4	Day 7	Day 10	AFB_1_ (ng g^−1^)
Control	-	-	-	14.24 ± 0.23 ^a^	-	-	-	-	n.d.
PCL-0	3.51 ± 2.48 ^b^	0.51 ± 0.36 ^b^	0 ^a^	13.65 ± 0.48 ^a^	3 ± 1.41 ^c^	1.47 ± 1.20 ^c^	0.88 ± 0.72 ^c^	0.88 ± 0.62 ^c^	n.d.
PCL-PN90	5.26 ± 0.00 ^b^	0.51 ± 0.36 ^b^	0 ^a^	13.77 ± 0.47 ^a^	9 ± 1.41 ^bc^	1.96 ± 0.69 ^c^	0.88 ± 0.72 ^c^	0.66 ± 0.54 ^c^	n.d.
PCL-L1	19.30 ± 2.48 ^a^	5.38 ± 1.38 ^a^	0.86 ± 0.61 ^a^	13.26 ± 0.30 ^a^	27 ± 1.41 ^a^	24.51 ± 1.39 ^a^	22.51 ± 1.80 ^a^	27.41 ± 1.12 ^a^	n.d.
REO-PN90	15.79 ± 0.00 ^a^	3.85 ± 1.09 ^ab^	0.86 ± 0.61 ^a^	13.22 ± 0.53 ^a^	14 ± 5.10 ^b^	14.22 ± 1.83 ^b^	7.60 ± 0.83 ^b^	17.32 ± 0.82 ^b^	n.d.

MGI (%) was calculated relative to the untreated control at each sampling day. AFB_1_ was measured at the end of incubation (day 7 at 25 °C; day 10 at 40 °C). AFB_2_, AFG_1_, and AFG_2_ were below the detection limit (LOD = 0.01 ng g^−1^) in all samples; n.d. = not detected. Values are expressed as mean ± SD. Different lowercase letters within each column indicate significant differences among samples (*p* < 0.05).

## Data Availability

The original contributions presented in this study are included in the article. Further inquiries can be directed to the corresponding author.
